# Assessment of acute and chronic pain in canine internal medicine—what is missing in our diagnostic toolbox?

**DOI:** 10.3389/fvets.2025.1614403

**Published:** 2025-11-26

**Authors:** Annina N. Sander, Sarah Storz, Panagiotis G. Xenoulis, Romy M. Heilmann

**Affiliations:** 1 Small Animal Practice Dr. Strauß, Halle a. d. Saale, Germany; 2 Department for Small Animals, College of Veterinary Medicine, University of Leipzig, Leipzig, Germany; 3 Veterinary Associates Elmshorn, Elmshorn, Germany; 4 Department of Clinical Sciences, College of Veterinary Medicine, University of Thessaly, Karditsa, Greece

**Keywords:** chronic enteropathy, enteritis, evaluation, pain scores, pancreatitis, severity, quality of life

## Abstract

Perceiving pain is a protective mechanism among all animal species. It involves sensing pain and experiencing the associated discomfort, aversion, and negative emotions. These aspects are particularly complex and potentially impossible to quantify as a multidimensional and subjective experience. Pain can be communicated in various ways. However, the inability to verbally communicate this experience remains a challenge to assess and quantify pain in dogs, as there is no gold standard. Species- and animal-specific factors (e.g., age, breed, clinical condition, or anxiety) also need to be considered. Several pain scales have been established in veterinary medicine to improve analgesic, medical, and/or surgical treatment and provide additional prognostic and/or diagnostic information. These scales focus on assessing behavior and objective physiological parameters (e.g., heart rate, systemic blood pressure, mydriasis), which are not specific and can be affected by large inter-individual variation. Addressing this challenge in canine acute and chronic pain management requires accurate and effective pain scoring systems that work reliably with the underlying condition. Particularly for internal medicine conditions, more work is needed in the future, as there are no specific tools available that might improve diagnosis and treatment. This article provides a brief overview of the current knowledge about acute and chronic pain assessment in dogs, available pain scales, which are based on subjective assessment, and the limitations and challenges of using these tools in clinical practice. It offers perspectives for novel avenues and future applications for clinical pain scales and individualized pain assessment in canine internal medicine.

## Definition of pain

The perception of pain is a common trait and protective mechanism among all animals and humans (1). Both physiological and behavioral evidence indicate that animals not only sense pain but also experience the associated discomfort, aversion, and negative emotions (1). Though this was historically questioned, it led to developments in pain management with many parallels to those of human infants. Today, the emotional health of animals remains one of the most difficult challenges faced in veterinary science and animal welfare (2).

The emotion of pain, as a complex and abstract construct, is currently impossible to quantify as it presents a multidimensional and subjective experience and is not directly measurable in animals due to their inability to self-report (3, 4). Given that pain is an inherently individual sensation, the intensity of pain is not measured directly, but rather inferred from the form and magnitude of its expression, meaning the impact of pain is measured (1, 2). According to the International Association for the Study of Pain (IASP), pain is *“*[…] *an unpleasant sensory and emotional experience associated with, or resembling that associated with actual or potential tissue damage* […]” (4). Because pain is an individual perception that is affected and modulated by many factors of biological, psychological, and social origin, there are various ways of communicating pain, and the absence of verbal communication regarding the experience of pain does not preclude its existence in either humans or non-human animals (4).

Although the delineation between acute and chronic pain is not clearly defined, it is conventionally based on an arbitrary time criterion, whereby pain present for more than 3 months is regarded as chronic (5). Unlike acute pain, which always represents an adaptive physiological response to a potentially harmful stimulus, chronic pain is commonly considered a distinct (maladaptive) condition or syndrome. It arises from the maladaptive processing of pain due to dysfunctional neurological transmission and lacks any physiological benefit. An experience causing pain invariably leads to an increased sensitivity to perceive pain in the affected body region. Consequently, normally innocuous stimuli may develop into an experience of pain over time. This can result in hyperalgesia—an exaggerated and prolonged response to a noxious stimulus—and allodynia—a pain response to a weak, normally harmless stimulus. Both hyperalgesia and allodynia arise from peripheral and central sensitization (5).

## Detection of pain and clinical goals

The assessment and quantification of pain in animals is highly challenging, as there is no gold standard to measure pain and its effects (1, 5, 6). The 2022 World Small Animal Veterinary Association (WSAVA) Global Pain Management Guidelines (1) suggest using behavioral signs and characteristics as the basis for recognition and assessment of pain. Age, breed, demeanor, type and duration of pain, underlying clinical condition, and the presence of any additive stressors such as anxiety might influence the species-specific or even animal-specific expression of pain (1). Using validated pain scoring systems by trained veterinary staff and rarely by capable owners is a recommended approach to establish a consistent way of measuring and monitoring pain (1).

In veterinary medicine, several different pain scales have been developed and validated over the past years. Most of these pain scales do not focus on measuring pain itself but the impact pain has on the animal’s behavior and objective physiological parameters. Physiological data such as tachycardia, tachypnea, systemic blood pressure, mydriasis, rectal temperature, and plasma cortisol and inflammatory cytokine (e.g., interleukin [IL]-2, IL-10) concentrations have proven to be ineffective as specific indicators of acute or chronic pain because these variables are neither sensitive nor specific to detect the effects of pain and are also altered by anxiety, fear, metabolic conditions, anemia, and various inflammatory and neoplastic processes (6–8). Quantifying these parameters is also not specific to identifying their cause or change, as they are highly subjective and show large inter-individual variation among animals of different species and even within the same species (9). Historically, pain measurement in human and veterinary medicine has focused on intensity, whereas the contemporary approach emphasizes the emotional or affective dimension, reflecting the unpleasant experience of pain demonstrated by changes in facial expression and behavioral cues (1, 10, 11). These include altered demeanor, aggressiveness, submissiveness, fearfulness, restlessness, lethargy, changes in activity, inquisitiveness, vocalization, self-mutilation, appetite, drinking, urination, grooming, and social behavior (10). The value of applying any pain scale to a specific patient is to improve analgesic, medical, and/or surgical treatment and to provide additional prognostic and diagnostic information (6, 12, 13). These pain scales are a means to improve the well-being of animals during the recovery phase, ensuring the patient’s pain does not remain unrecognized and untreated or undertreated (6). The behaviors displayed by various species, including dogs, however, can differ substantially from those observed in humans and, in certain situations, may be completely obscured by the animal’s response to being watched while experiencing fear, stress, and/or anxiety (2). This lack of unequivocal pain indicators can result in the false assumption that the animal is not experiencing relevant levels of pain. Addressing this challenge in pain management requires accurate and effective pain scoring systems that work reliably with the underlying acute or chronic condition of the patient (2, 9).

## Pain scales in veterinary medicine

Among such subjective and semi-objective scoring systems are the Preemptive Scoring System, the Visual Analog Scale (VAS), the Simple Descriptive Scale (SDS), and the Numerical Rating Scale (NRS) (14). The Dynamic Interactive VAS (DIVAS) was developed to assess pain following routine elective surgical procedures, such as ovariohysterectomy. DIVAS involves the evaluation of changes in facial expression, behavior, and vocalization using a 0–100 (mm) scale (9). These scoring systems can be used by both veterinary staff and patient owners, presenting simple but still inaccurate ways to quantify the intensity of pain and/or its impact. These surgical scales also tend to be affected by considerable inter-observer variability, and their unidimensional approach may fail to fully capture the complexity of the development and experience of pain (3, 6).

**Figure 1:**
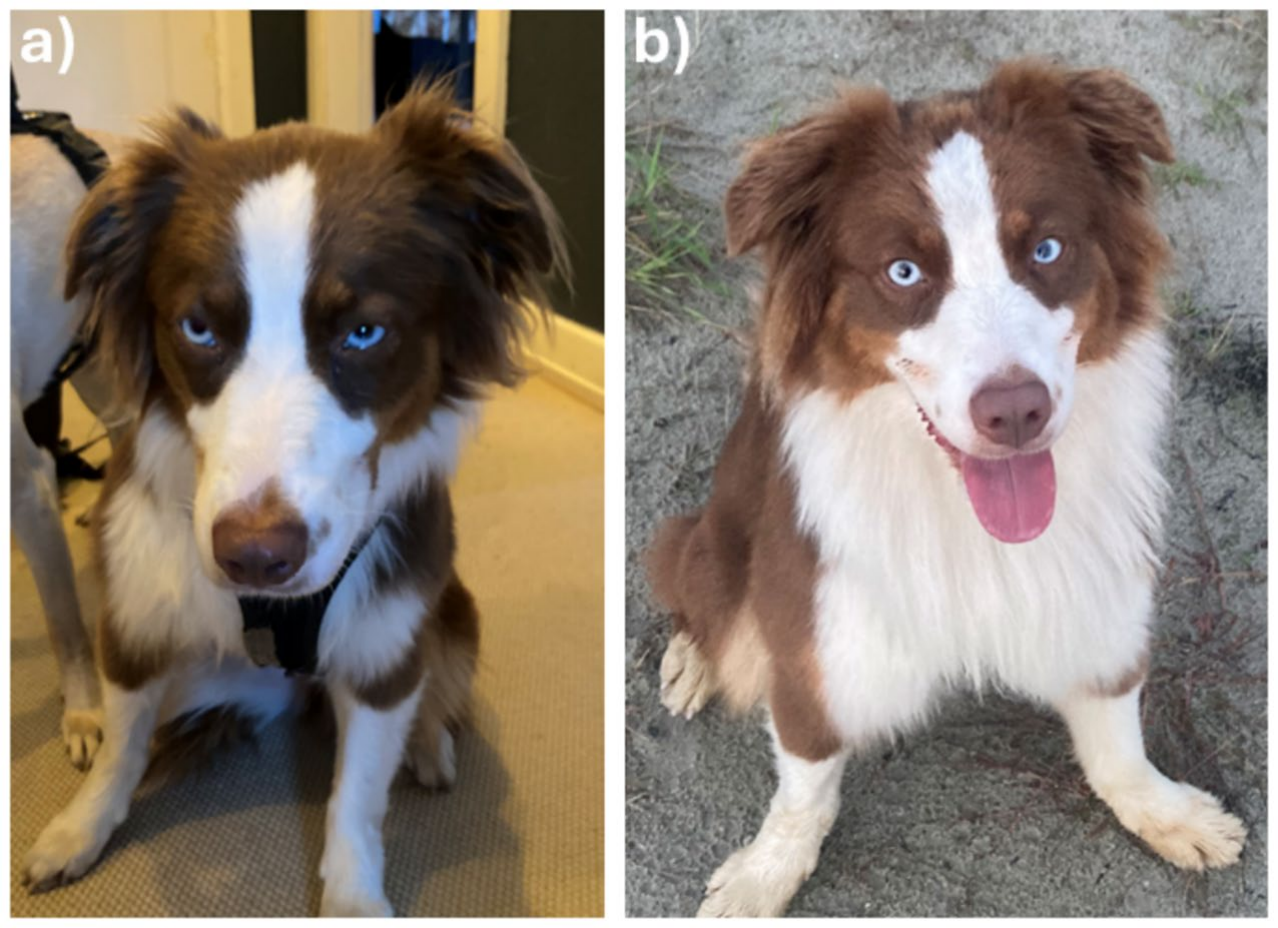
Facial expression as a behavioral aspect in the assessment of pain in dogs. Shown are **(a)** a dog with chronic enteropathy experiencing nausea and abdominal discomfort during a disease flare-up, and **(b)** the same dog after reaching disease remission. Notice the posture of the ears pointing toward the side, the oval shape of the eyes resulting in a “sad” or “tired” appearance, the downward and away gaze, and a tightly closed mouth **(a)** as opposed to the dog’s presentation in **(b)**.

**Figure 2:**
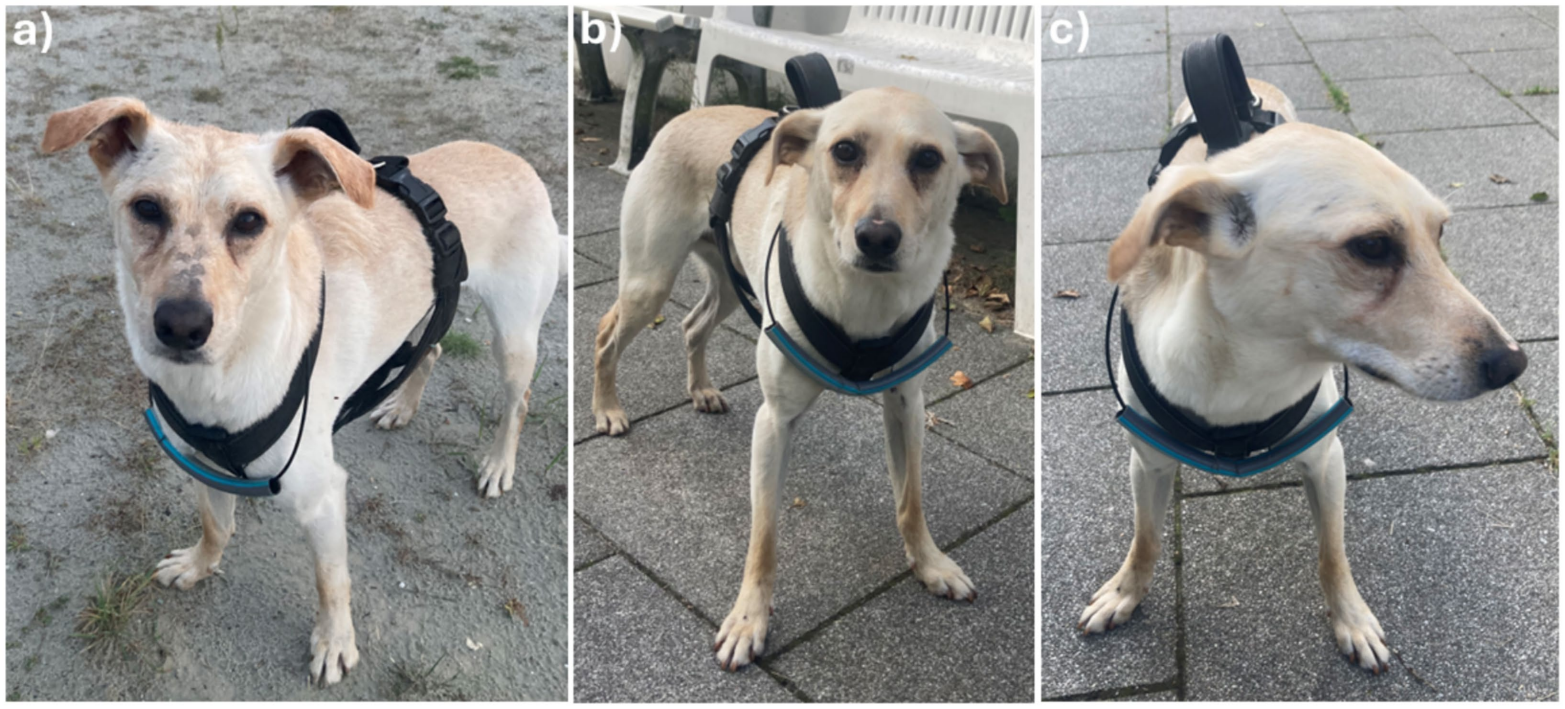
Behavioral cues to assess pain in dogs. In contrast to **(a)** the normal expression of this dog, behavioral cues shown during a chronic enteropathy flare-up include **(b)** a change in posture, lowered and backward pointing ears, smooth forehead, and increased tension of the periorbital muscles, and **(c)** an away gaze and tension of the facial muscles as well as bristled whiskers.

Grimace scales were developed as a species-specific clinical tool to identify pain in several species by evaluating similar regions or features. They are simple to use and therefore practicable for both trained staff but also owners. For example, the equine’s “face of pain” comprises characteristics such as an asymmetric carriage of the ears, increased tension of the orbital muscles, and raised lips (9). Although dogs can exhibit changes in facial expression patterns related to pain—such as changes in the position of the ears (Figures 1a,b) and head and tension of the muscles around the nose, mouth, and eyes, including the inner brow raiser, blink, and lip corner puller (Figures 2b,c)—there is currently no universally accepted grimace scale for dogs. This is primarily due to significant inter-breed variations in this species (9, 11). With dogs being domesticated and living in close contact with humans for thousands of years, they would appear as the species interpreted by humans much better than any other species (7). This, in turn, would make dogs the best candidates for the development of instruments to detect and measure the impacts of both acute and chronic pain using subjective judgment (10).

Several pain assessment scales are widely used to evaluate dogs experiencing acute or chronic clinical pain (Table 1) (15, 16, 18–23). These include the University of Melbourne pain scale (UMPS) (17), the Colorado State University canine acute pain scale (CSU-CAPS) (16), the Glasgow composite measure pain scale (CMPS) (24), and its abbreviated version, the short form of the CMPS (CMPS-SF) (15). Only some of these acute and/or surgical pain scales include the recognition of physiological and biological alterations. Few scales integrate facial expression and behavioral changes, physiological parameters, measurement of biochemical mediators such as plasma cortisol concentration, atypical vocalization, and general body functions such as food and water intake or body weight changes (7, 14).

**Table 1:** Examples of scales established to evaluate acute pain (*n* = 3) or chronic pain (*n* = 6) in dogs.

Scoring system	Target condition	Primary evaluator	Level of scoring	Primary utility	Validity	Assessed criteria
CMPS-SF (Glasgow short form scale) (15)	Acute pain	Veterinarian, veterinary support staff	Moderate	Monitoring	Partially validated	Vocalization, attention to a wound, mobility, response to touch, demeanor, posture/activity
CSU-CAPS (Colorado acute pain scale canine) (16)	Acute pain	Veterinarian, veterinary support staff	Simple	Monitoring	Not validated	Behavioral signs, response to palpation, body tension
UMPS (University of Melbourne pain scale) (17)	Acute pain	Veterinarian, veterinary support staff	Moderate	Monitoring	Partially validated	Heart and respiratory rate, rectal temperature, response to thoracic auscultation and body palpation, activity level, emotional state, posture, vocalization
LOAD (Liverpool osteoarthritis in dogs) (18)	Chronic pain, osteoarthritis	Owner or caretaker	Simple	Monitoring	Validated	Total of 13 ability- and mobility-associated criteria
CBPI (Canine brief pain inventory) (19)	Chronic pain, osteoarthritis	Owner or caretaker	Simple	Monitoring	Validated	Pain intensity, activity, mobility, QoL
SNoRE (sleep and nighttime restlessness evaluation) (20)	Chronic pain, osteoarthritis	Owner or caretaker	Simple	Monitoring	Validated (version 3.0)	Behavior during sleep
HCPI (Helsinki chronic pain index) (21)	Chronic pain, osteoarthritis	Owner or caretaker	Simple	Monitoring	Sparsely validated	Mobility, posture, mood, general behavior
CSOM (CLIENT-specific outcome measures) (22)	Chronic pain, osteoarthritis	Owner or caretaker	Moderate	Monitoring	Partially validated	Total of 6 ability- and mobility-associated criteria
COAST (Canine osteo-arthritis staging tool) (23)	Chronic pain, osteoarthritis	Owner or caretaker, veterinarian	Moderate	Screening	Not validated	Mobility, QoL, BCS, orthopedic examination findings, radiographic evidence of osteoarthritis lesions

BCS, body condition score; QoL, quality of life.

The University of Melbourne pain scale (UMPS) is a multidimensional scale used to assess the effects of pain in dogs by veterinarians and other animal care professionals (17). This acute pain scale evaluates six variables: physiological parameters (heart and respiratory rate, rectal temperature), behavioral responses to thoracic auscultation and body palpation, activity level, emotional state, posture, and vocalization (17). Though it is not designed to be used on sedated animals, it is generally a precise clinical tool offering a high level of specificity and sensitivity for the detection and quantification of alterations that can be caused by acute pain (7, 17).

The Colorado State University Feline and Canine Acute Pain Scale (CSU-CAPS) is suitable for evaluating pain in both dogs and cats (16). Initially created as an educational tool in the clinical setting, it is easy to use as it provides five different categories considering physiological and behavioral components, response to auscultation and palpation, and rigidity of the body. Using predefined criteria, each category is assigned a score between 0 (normal) and 4 (severely abnormal). The cumulative CSU-CAPS score is calculated as the sum of the individual scores for each category and is used to assess the overall pain level of the patient. This acute surgical pain scale is primarily used to estimate an animal’s need for analgesia (7, 16, 25).

The pain scale currently used most frequently in small animal medicine is the Glasgow composite measure pain scale (CMPS) (24), along with its modified short form (CMPS-SF) (15). The original CMPS included the evaluation of seven behavioral variables: demeanor and response to people, posture, mobility, activity, response to touch, attention to the painful area(s), and vocalization (24). This score evaluates acute pain levels in dogs in the hospital setting by observation and interaction with the animal through veterinary professionals. However, a limitation might be the lack of numeric scoring and, as a result, limited comparability (24). The CMPS short form (CMPS-SF) presents an easier and faster-to-use numerical tool in a clinical setting. It evaluates six different variables based on descriptive items: vocalization, attention to wound, mobility, response to touch, demeanor, and posture/activity (15). By ranking these items in ascending order of pain intensity, the observer selects the item in each category that most accurately represents the dog’s behavior. Summing up the ranked scores, the maximum pain score is 24 (if all criteria are evaluated) or 20 if mobility cannot be assessed (15).

## Challenges with the clinical use of pain scales

The existing pain scales work reliably for routine clinical use, but some limitations need to be considered. All of these schemes rely heavily on observation, which might result in evaluator errors or biases. Studies show that sporadic monitoring, possibly by more than one veterinary staff member, can lead to significant variability among observers for the use of pain scales (6, 9, 14, 26). Therefore, analyses of pain score data in dogs must account for inter-observer variability when multiple observers are involved in the assessment of pain, as well as intra-observer reliability when these assessments are performed by the same observer (14, 26). Similar to studies assessing pain through lameness evaluation in other species, such as in cattle and showing large intra- and interobserver variability (27), a study in dogs confirmed significant inter-observer variability (29–36%) among veterinarians using the simple descriptive, numerical rating, and VAS (14). Similar variations were reported for the CMPS-SF (28). The more frequently a pain scale is used, the greater the likelihood of individual modification, which may result in varying outcomes (3). In addition, gender and level of professional experience of the observer can affect the use of a pain scale and thus the associated inter-observer variability (29, 30). Although a dog’s facial and body expressions provide valuable information about its emotional status, additional factors may also have the potential to affect the human perception of pain (8). For example, acute stress can override pain responses and even lower the tolerance of pain perception. This might compromise an accurate assessment of pain, especially if the dog experiences stress during the evaluation (e.g., during hospitalization) (8). Pharmacological intervention (e.g., administration of sedatives, analgesics, and/or anesthetics) can also significantly affect the manifestation of pain-related behaviors in dogs (13).

Furthermore, indicators of pain may be masked by behaviors characteristic of the species being observed, such as a dog wagging its tail and greeting an observer at the cage door despite being in significant pain (6, 8). Another challenge is the significant visual and anatomical variability among dogs and dog breeds, which makes it nearly impossible to standardize facial expressions across all canine breeds.

## Current needs and future applications for clinical pain scales

Medical conditions in which the presence and severity of pain experienced by the affected animal are particularly difficult to assess include internal medicine diseases, such as certain acute or chronic (recurring or persisting) gastrointestinal, urogenital, and other visceral diseases (e.g., acute or acute-on-chronic pancreatitis, cholecystitis, flares of chronic inflammatory enteropathy, glomerulonephritis, or idiopathic cystitis) (31). Behavioral indications of particularly chronic pain, such as the loss of normal behaviors and the emergence of new or abnormal ones, can be very subtle and easily missed by veterinary staff and even the owners (31). Most of the pain scales established and validated in veterinary medicine consider only acute perioperative and postoperative pain (2, 3, 6, 7, 9, 14, 15, 17, 24, 25). However, a pain scale that also accounts for the evaluation of other types of pain, such as acute pain and/or distress caused by acute pancreatitis (Figure 1) and other more chronic internal medicine diseases, such as chronic recurrent gastroenteritis (Figure 2), is currently lacking in veterinary medicine. This gap in the clinician’s diagnostic toolbox creates a critical issue: applying a standard pain scale developed for post-surgical pain to cases of acute non-surgical pain or either persistent or recurrent chronic pain in dogs is likely to underestimate the presence or severity of pain. As a result, pain scores determined using these scales may fall short of establishing an adequate threshold to initiate appropriate analgesic treatment for these conditions (6, 7).

### An internal medicine example—integrating pain assessment in the management of canine acute and acute-on-chronic pancreatitis

Acute pancreatitis is a disease that is often associated with severe abdominal pain and discomfort in dogs and, therefore, clinical hospitalization to optimize treatment and patient monitoring (e.g., for possible complications) (32). Although a single pathognomonic clinical sign does not exist for acute pancreatitis in dogs, the clinical presentation often includes anorexia, vomiting, weakness, and abdominal pain, which in only very severe cases can be presented by assuming a “prayer-position” (forequarters on the ground, hindquarters up) (1, 7, 32–34). However, such signs of acute pain are not nearly as obvious in most affected dogs. Diagnosing acute pancreatitis is challenging, and a combination of diagnostic tools is necessary to establish a final diagnosis (32, 33, 35). The Modified Canine Activity Index (MCAI) is a scoring system that helps to assess the severity of pancreatitis and might improve the evaluation of the patient’s clinical outcome (35, 36). This mainly subjective clinical score does not correlate perfectly with the measurement of serum biomarkers and, therefore, might not have the desired accuracy to assess the clinical progression of acute pancreatitis in dogs (36). However, the seven variables included in the MCAI (activity, appetite, vomiting, cranial abdominal pain, dehydration, stool quality, and blood in stool) could still be useful in further refining the MCAI scoring system and developing an optimized scale for pain assessment in dogs with acute or acute-on-chronic pancreatitis.

Alleviating pain in conditions such as acute pancreatitis is not only a professional responsibility of the attending veterinarian but is also a crucial factor in achieving successful patient outcomes and strengthening the veterinarian–client–patient relationship (37). Quality of life (QoL) measurements in dogs are structured tools used to objectively assess an animal’s overall well-being, which includes its physical condition, behavioral patterns, and any effects of disease or medical treatment (38). Several health-related QoL measurement tools exist to assess a dog’s status of well-being in a generic or disease-specific condition (e.g., chronic gastrointestinal diseases, Table 2) (39–59). QoL is not only defined by the absence of disease and disability, but also includes subjective and individual experiences such as the need for satisfaction, sense of control, social relationships, the extent of physical or emotional discomfort, and management of stress, and therefore is highly affected by acute and chronic pain (39, 60). While QoL tools provide a broad overview of an animal’s general well-being, health-related QoL (HRQoL) instruments offer a more nuanced understanding of how specific health conditions and treatments impact daily functioning and comfort (39–48, 51–59). Veterinarians and owners together can best evaluate a dog’s HRQoL to assist in the treatment of diseases such as diabetes mellitus, chronic kidney disease, cancer, and heart disease. This integrative approach also allows for potential euthanasia decisions if HRQoL has declined below a critical minimum. Although all (HR) QoL scales are designed differently and interpreted individually, the focus of all (HR) QoL scales is the evaluation of three main aspects: activity level, appetite, and the desire to interact socially (38). However, these scales are limited to the assessment of pain-specific behavior, which shows the importance of the need for developing and validating additional acute and chronic pain measurement scales or refining and extending currently available acute surgical pain scales, especially for conditions associated with severe pain like acute pancreatitis.

**Table 2:** Examples of health-related quality of life (QoL) tools established to evaluate pain in dogs modified from Reid et al. (10).

Reference	Application	Target condition	Items	Comments
Lavan (39)	Generic	Overall health status	15	Simple scoring system, targets healthy dogs to monitor changes over time
Wojciechowksa et al. (40)	Generic	Several conditions	27	Simple scoring system, nonphysical aspects of QoL
Reid et al. (41)	Generic	Several conditions	46	Complex score (includes vigor, pain, anxiety, stress), web-based format
Reid et al. (42)	Generic	Several conditions	22	Complex score (includes activity, comfort, balance)
Fefer et al. (43)	Generic	Several conditions	17	Complex score (includes vitality, mobility, pain, companionship)
Lavan et al. (44)	Generic	Several conditions	34	Complex score (includes several domains)
Marchetti et al. (45)	Disease-specific	Chronic enteropathy	5	Simple scoring system, includes other assessment scales (VAS, CCECAI, LAPS, C-BARQ)
Díaz-Regañón et al. (46)	Disease-specific	Chronic enteropathy	14	Complex score (includes dog- and owner-related items)
Wells et al. (47)	Disease-specific	Canine allergic dermatitis	26	Moderately complex scoring system, includes treatment satisfaction
Gildea et al. (48)	Disease-specific	Canine osteoarthritis	26	Simple scoring system, includes both dog and owner QoL as well as owner treatment satisfaction
Freeman et al. (49)	Disease-specific	Canine cardiac disease	17	Simple scoring system, FETCH questionnaire

C-BARQ, canine behavioral assessment and research questionnaire; CCECAI, canine chronic enteropathy clinical activity index; FETCH, functional evaluation of cardiac health; LAPS, Lexington attachment to pets scale; QoL, quality of life; VAS, visual analog scale.

## Future outlook on pain assessment in veterinary medicine

If pain is considered a completely subjective experience, reliable measurement should aim to assess that individual perception. Current studies show that video recordings could provide an unbiased view of the pain-related behavior of dogs (8, 11, 61). If the observer is not recognized or hidden, dogs may be less likely to mask their emotions, even if they are in an unfamiliar environment. As an example, video recordings are likely to capture facial expressions and movements of the ears associated with pain, such as tensing of the masseter muscle and downward rotation of the eyes (8). If the intensity of pain is high, other behavioral changes or patterns, such as aggression or an opposite response (depression, fear, vocalizations, reduced interaction, restlessness, and anorexia) may be displayed (7–9). Another possibility to assess pain-related changes in movement and activity outside of a clinical setting could be activity monitors (62). Due to their small size, minimal invasiveness, mobility, and ability to collect objective data remotely, these devices represent a promising diagnostic tool for use in the animal’s natural environment. In line with this, interpretation by a well-trained and highly skilled professional, potentially with the aid of digital health and artificial intelligence tools, can be expected to further reduce the risk of subjective misjudgments and help with the provision of accurate results, leading to optimized and individualized patient management and monitoring. Such an integrated approach may lead to an improved assessment of pain in dogs with acute or chronic gastrointestinal diseases, such as recurrent episodes of pancreatitis, with the potential to better tailor treatment to the individual patient and enhance patient care and outcomes. Emerging technical approaches for pain assessment in dogs that remain to be rigorously validated further include the Parasympathetic Tone Activity (PTA) index (63, 64) and infrared pupillometry (65–68).

Recognizing that animals are capable of experiencing pain means that veterinarians have a responsibility and obligation to reduce its occurrence through both prevention and treatment. This task entails an appropriate pain management strategy, which ideally is titrated to the individual needs of the patient. Decades ago, veterinarians had access to a much more limited range of analgesics and may have been more likely or more often overlooked or underestimated pain and its significance due to the restricted ability to manage pain effectively (69). Such an approach has become hard to justify, given the broader understanding of acute and chronic pain as a crucial component of patient management in veterinary medicine and the emphasis placed on relieving pain in the clinical practice setting (69).

## Conclusion

Understanding pain as an individual experience originating from a variety of possible sources and leading to individual perceptions and responses in animals is expected to pave the way to further efforts in pain recognition and quantification, not limited to the evaluation of post-surgical and orthopedic patients. However, evaluating pain in animals under clinical conditions remains challenging and prone to bias or misjudgment. Awareness of the limitations associated with developing a comprehensive chronic pain scale for veterinary internal medicine patients is important, as this task will be difficult and demanding, but indispensable to improving the quality of life of veterinary patients in the future. It is to be emphasized that these efforts will specifically need to integrate individual differences in the expression of pain in dogs as a species with highly complex social behaviors.

## Data Availability

The original contributions presented in the study are included in the article/supplementary material, further inquiries can be directed to the corresponding author.
